# University of Buffalo

**Published:** 1898-05

**Authors:** 


**A Monthly Review of Medicine and Surgery.**

*EDITORS:*

THOMAS LOTHROP, M. D. - - WM. WARREN POTTER, M. D.

All communications, whether of a literary or business nature, should be addressed to the managing editor: 284 FRANKLIN STREET, BUFFALO, N. Y.

VOL. XXXVII. MAY, 1898. No. 10.

### UNIVERSITY OF BUFFALO.

### ANNUAL COMMENCEMENT OF MEDICAL, PHARMACAL AND DENTAL DEPARTMENTS.

THE University of Buffalo observed the fifty-second anniversary of its medical department with its annual commencement exercises on Tuesday, April 26, 1898, at which 166 graduates in the three departments of medicine, pharmacy and dentistry received their diplomas. The curators of the university met in the forenoon to listen to the questions that the faculty propounded to the classes in their presence, and to formally recommend the candidates to the council for degrees.

### ALUMNI ASSOCIATIONS.

The alumni association of the medical department held its twenty-third annual meeting in Alumni Hall of the college building in the afternoon of commencement day.

The president, Dr. D. A. Currie, '64, of Englewood, N. J., called the meeting together promptly at two o'clock. The members in large numbers from different sections of the country registered their attendance. Among the older alumni were Drs. A. G. Ellenwood, '48, of Attica; C. C. Wyckoff, '48, of Buffalo; Thomas D. Strong, '51, of Westfield; William Warren Potter, '59, of Buffalo; George M. Palmer, '65, of Warsaw; R. J. Menzie, '66, of Caledonia.

The election of officers for the ensuing year resulted as follows: President, Dr. Z. J. Lusk, of Warsaw; first vice-president, Dr. William Warren Potter, of Buffalo; second vice-president, Dr. M. A. Veeder, of Lyons; third vice-president, Dr. E. G. Hoitt, of Marlborough, Mass.; fourth vice-president, Dr. W. R. Campbell, of Niagara Falls; fifth vice-president, Dr. A. W. Hinkly, of Rochester; permanent secretary, Dr. E. L. Frost; recording secretary, Dr. N. V. Chappell; treasurer, Dr. H. U. Williams. On the board of trustees, Dr. D. A. Currie was elected to succeed Dr. Joseph Fowler, whose term expired. The trustees are Drs. Julius Wenz, of Lancaster; H. P. Trull, of Williamsville; C. A. Wall, William C. Phelps, of Buffalo; and D. A. Currie, of Englewood, N. J. Dr. A. T. Kerr was elected a member of the executive committee, which consists of the following-named: Drs. Albert T. Lytle, Grover W. Wende, A. T. Kerr, Z. J. Lusk and John Parmenter, the last two *ex officiis*.

After the business session was terminated, the president, Dr. Currie, delivered his address, entitled, Living and dying; their physics and psychics. This scholarly address attracted close attention and received, as it merited, enthusiastic applause. It is impossible to do justice to it in a brief abstract. In his opening paragraph he referred to the mystery of life and spoke of the little the researches of science had done toward the solution of the mystery. The length of human life was referred to, both of the antediluvian age and the present.

He said: "Before the flood the physical constitution was complete and its vital functions were perfect. We cannot solve the problem of the long lives of men of that period. They retained health and vigor of the body through many centuries, and, as far as we know their history, we have no intimation that sickness was known. Considering man as he now is, the most distinguished physiologists of our times are agreed in the opinion that three score years and ten is the average and 100 years is the extreme limit of life." The manner in which death acts and its signs from a medical viewpoint were discussed at length.

Dr. Currie illustrated his statements by reference to authorities in medical science and also the literary world, quoting liberally from both. In regard to the sensations at the time of death, Dr. Currie did not hold the opinion that they are painful. He said: "We are not necessarily born to suffer. All natural conditions, more or less, are sources of pleasure. Our physiological nature is such that the last phenomena in natural death are the same as those of sleep. The difference between sleep and the act of dying is, one is temporary, the other is for all time."

In closing, the speaker touched upon the spiritual aspect of the end of life, saying: "The union of the body and soul is a mystery. When we see the bond finally and completely severed, we stand in silent wonder."

The scientific part of the program was next taken up and Dr. Daniel Lewis, of New York, president of the state board of health, introduced for consideration the subject of cancer, with a short but pointed and exceedingly well-presented address, entitled, The precancerous stage. We hope to publish his remarks in full in an early edition of the JOURNAL. Dr. Roswell Park discussed the subject from the viewpoint of infection and Dr. Woods Hutchinson generalised in a few remarks, distinguished for their graceful rhetoric.

Dr. E. M. Houghton, of Detroit, then presented a paper, entitled, Antitoxic serum, which was an admirable setting forth of the present status of serum therapy. The remainder of the program, as published in the announcement, is as follows: *Diphtheria:* Community sanitation and laws, Dr. Frank A. Jones, Rochester; discussion, Dr. Walter D. Greene, Buffalo. Personal sanitation, Dr. John Foote, Lockport; discussion, Dr. Henry R. Hopkins, Buffalo. Bacteriology, Dr. George M. Palmer, Warsaw; discussion, Dr. William G. Bissell, Buffalo. Morbid anatomy, Dr. Melville C. Follett, Olean; discussion, Dr. Herbert U. Williams, Buffalo. Symptoms, Dr. Robert J. Menzie, Caledonia; discussion, Dr. DeLancey Rochester, Buffalo. Diagnosis, Dr. S. R. Cochrane, Medina; discussion, Dr. Charles G. Stockton, Buffalo. Medical treatment, Dr. Frederick F. Hoyer, Tonawanda, Dr. Rollin T. Rolph, Dunkirk; discussion, Dr. Irving M. Snow, Buffalo. Surgical treatment, Dr. Charles H. Richmond, Livonia, Dr. Selden J. Mudge, Olean; discussion, Dr. George F. Cott. Buffalo.

Owing to the lateness of the hour it became necessary to modify the foregoing schedule, omitting some of its details. When the hour for adjournment came it was the general sentiment of those present that the meeting had been most interesting and profitable.

The annual meeting of the alumni of the dental department was held Tuesday afternoon, April 26th, at the college. Dr. T. G. Gibson was elected president, Dr. William Bartlett, vice-president, and Dr. H. T. Squire secretary and treasurer. Immediately following the conclusion of the commencement exercises of the class, the alumni gathered around the banquet table at the Iroquois.

The alumni association of the department of pharmacy held its annual meeting Wednesday, April 27th, at the college. There was a business meeting in the morning and in the afternoon W. C. Alpero, of New York, delivered an address. In the evening the association held its banquet at the Genesee.

### THE EVENING AT MUSIC HALL.

The graduating exercises began at Music Hall at 8 o'clock. The seats in the center had been reserved for graduates, who marched to their appropriate places, clad in caps and gowns, to tuneful strains from Kuhn's orchestra. The faculty and council took their seats upon the stage and the Rev. O. P. Gifford opened the exercises with prayer. Dr. John Parmenter, secretary of the medical faculty, presented the class; Dr. Matthew D. Mann, dean of the medical faculty, administered the Hippocratic oath, and then the chancellor, Hon. James O. Putnam, in well-formulated words, conferred the degrees. The dean announced that the thesis of Julius Samuel Berkman, on the subject of Addison's disease, and also that of Nelson W. Wilson, on Sunstroke, had been found worthy of honorable mention and were also to be bound and placed in the college library for reference. Theses of the following-named were mentioned: Joseph L. Howell, Richard J. Gould, Gifford Truesdell, Clifford R. Orr and Homer J. Knickerbocker. Thomas H. McKee, Abram L. Weil, Ray K. Barry and Edith Winifred Stewart secured averages above 90 per cent. during their course of study.

*Class in Medicine.*—Alexander Allan, Buffalo, N. Y., Robert Marion Andrews, Bergen, N. Y.; M. Louis Bacon, Buffalo, N. Y.; Frederick C. Ballard, Centerville, N. Y.; Ray K. Barry, Willink, N. Y.; A. Austin Becker, Rochester, N. Y.; Julius Samuel Berkman, Bath, N. Y.; William J. Bott, Buffalo, N. Y.; Charles Bigelow Braman, Buffalo, N. Y.; Clayton A. Button, Holland, N. Y.; Annie May Cheney, Franklinville, N. Y.; John C. Cleary, Clyde, N. Y.; Georgia Cruickshank, Buffalo, N. Y.; Arthur I. Eccleston, Kent, N. Y.; Frederick W. Filsinger, Buffalo, N. Y.; John Robert Bruce Glasgow, Montezuma, N. Y.; Scott D. Gleeten, Waterford, Pa.; Richard J. Gould, Lockport, N. Y.; Herbert I. Harris, Buffalo, N. Y.; John L. Hazen, Penn Yan, N. Y.; Joseph Lewis Howell, Newton, N. J.; H. Francis Hunt, Clark, N. Y.; Harry B. Huver, M. D., D. D. S., Williamsville, N. Y.; Edwin Will Janes, A. B., Randolph, N. Y.; Irving R. Johnson, St. Catharines, Ont.; John N. Jones, Erin, N. Y.; Henry Joslyn, Buffalo, N. Y.; Thos. A. Killip, Rochester, N. Y.; Homer J. Knickerbocker, Ph. G., Elba, N. Y.; Caroline Lichtenberg, Buffalo, N. Y.; Thomas H. McKee, Buffalo, N. Y.; Fred B. McKenney, A. B., Rochester, N. Y.; Jonathan Tamblyn Male, A. B., Beech Lake, Pa.; Monroe Manges, A. B., A. M., M. D., Buffalo, N. Y.; Gus A. C. Mannel, Rochester, N. Y.; Howard A. Maynard, South Butler, N. Y.; Allie Long Mitchell, A. B., East Aurora, N. Y.; William H. Mountain, Belfast, N. Y.; Charles H. North, Buffalo, N. Y.; Wilford L. Odell, Buffalo, N. Y.; Clifford R. Orr, Buffalo, N. Y.; Jacob S. Otto, A. B., Buffalo, N. Y.; Donald Parker, Stamford, Ont.; Albert Walter Phelps, North Evans, N. Y.; Alfred Percy Pierce, Buffalo, N. Y.; Charles Louell Preisch, Lockport, N. Y.; Charles Roemmelt, Elmira, N. Y.; Marie Cecelia Rotheram, Southport, Eng.; Edward Affleck Sharp, Buffalo, N. Y.; George A. Sloan, Buffalo, N. Y.; H. MacVicker Smith, Buffalo,

N. Y.; Edward A. Southall, Geneseo, N. Y.; Charles Wilson Southworth, A. B., Forestville, N. Y.; Floyd L. Spaulding, Howard, N. Y.; Peter A. Stadlinger, D. D. S., Buffalo, N. Y.; Edith Winifred Stewart, Hume, N. Y.; Walstein M. Tompkins, New York, N. Y.; Gifford Truesdell, Warsaw, N. Y.; Emory M. Wadsworth, Warsaw, N. Y.; Louis Talcott Waldo, Berkshire, N. Y.; Abram L. Weil, Buffalo, N. Y.; Nelson W. Wilson, Buffalo, N. Y.; Loretta E. Wooden, Rochester, N. Y.

Dr. John R. Gray, secretary of the faculty of pharmacy, presented the candidates in that department. Two members of the class, James A. Howland and Mark M. Minor, were entitled to the degree of master in pharmacy. Thirty-one members received the degree of graduate in pharmacy and three received certificates of examination which will entitle them to diplomas on their attaining the required age.

Nelson M. Wiegand won the William H. Peabody prize, having maintained the highest average in the class during the course. James A. Howland secured the advanced course prize. Frank T. Dewey, of the junior class, received the faculty junior prize. Honorable mention was made of the following: Nelson M. Wiegand, Mark H. Minor, Orlando M. Baker, Otto H. Salchow and James A. Howland.

*Class in Pharmacy.*—James Albert Howland, Mark Manchette Minor, Frank J. Babcock, Orlando Merriam Baker, John Henry Bradley, Frederick Henry Coon, Clarence Robert Cox, Franklin Seth Cushing, Gustave A. Gamenthaler, Fred Leroy Gibbs, Lawrence E. Green, Luke Frank Harvey, Arthur Homer Hennage, James Erwin Jones, George Burnett Kerr, Samuel Kavinoky, Peter Klingler, Merrick T. Marcy, Albert V. Mentz, Agnes Magelene Murray, Bartholomew E. Oats, William J. O'Shaughnessy, George B. Parkinson, Boris Reinstein, James Peter Rooney, Frederick A. Rudolph, Otto Henry Salchow, Ernest Frederick Slater, Charles Barton Skinner, Arthur Nelson Smith, Henry W. Veith, David George Wallace, Thomas Frederick Williams.

The following-named, having met all requirements, except that of age, received certificates of examination: Herbert Roy Edmonds, Reynold A. Janke, Nelson McKay Wiegand.

The class in dentistry was presented by Dr. A. P. Southwick, secretary of the dental faculty. Dr. W. C. Barrett administered the oath of the profession of dentistry. The honor roll, as read by Dr. Southwick, comprised those whose averages during their entire course of study had exceeded 90 per cent. They were Josephine H. Spillman, Grace G. Rankin, Miles M. Smith, Edward L. Schlottman, Clinton H. McCallum, Robert Murray and George M. Decker.

*Class in Dentistry.*—George Washington Averell, Edward Barber, Edgar Atheling Bartlett, Charles Wesley Borland, William Arthur Bostwick, Byron Alfred Brown, George Brown, Jacob Harry Brown, Charles Felix Buckland, Harry Dayton Burghardt, Egerton Bidwell Cays, Claude Robert Christopher, John

Emerson Cole, Arthur Herbert Consaul, Herschel Jacob Cull, Harry Washington Day, George Martin Decker, William Brown Dills, Kernon James Donahoe, John Lockhart Dudley, Moss Burton Eshleman, Raymond Reece Evans, Frank Leslie Frank, Katharine Magdelena Graf, Allen Lewis Green, John Nickels Hanna, Albert Devitt Heist, Harry Collins Howell, George Washington Jones, Albert Hugh Jung, Otto Charles Kalbfleisch, Henry Wilford Kitching, Charles Henry Laborn, Christian Albert Landel, Clarence Edward Lauderdale, Clinton Henry McCallum, Arthur Amzi Mix, Herbert Butler Morris, Robert Murray, Judson Hedden North, George Albert Northrup, William Wilton Paull, John Robert Quigg, Harry Budd Randall, Grace Greenwood Rankin, Howard Frank Redfield, Earnest Emerson Rice, Samuel Emerson Salisbury, Edward Leonard Schlottman, William Francis Sheahan, William Derwood Slacer, Miles Montaine Smith, Josephine Harriett Spillman, Louis Almon Squires, Ph. B., Ella Amanda Staley, George Stevenson, Duncan Stewart, Eli J. Sweet, Charles Dickinson Tefft, John Clarence Burroughs Tooke, George Carman Van Marter, Francis Harold Wallace, Louis Eidt Wettlaufer, Jay Leonard Wilson, Fred Arthur Woodman.

The Rev. Mr. Gifford addressed the graduates. His address was very interesting and he frequently was compelled to pause on account of the laughter his witty words provoked.

THE BANQUET.

The annual banquet of the alumni association was given at the Ellicott Club. At eleven o'clock the strains from Marcus's orchestra announced that dinner was served, whereupon the faculty, alumni and guests marched into the beautiful banquet hall of the Ellicott Club, and took their seats in groups at small tables that were handsomely decorated. At a large round table, decorated with red lights, yellow tulips and ferns, were grouped the president of the alumni, Dr. D. A. Currie, with Mr. Adelbert Moot and the Rev. Charles C. Albertson on his right and left respectively. Others at the table were : Dr. Daniel Lewis, of New York, Dr. Matthew D. Mann, Dr. H. M. Hill, Dr. Charles G. Stockton, Dr. Ernest Wende, Mr. John B. Olmsted, Dr. W. D. Greene, Dr. Delancey Rochester, Dr. H. G. Matzinger, Dr. H. U. Williams and Dr. William Warren Potter. The quality of the dinner was beyond criticism, and when coffee and cigars were served the speaking commenced. Dr. Currie acted as toastmaster, sending forth each speaker with a few bright and appropriate remarks. The following is the list of toasts and speakers : The University of Buffalo, Adelbert Moot, Esq.; State sanitation, Daniel Lewis, M. D.; Medical men and ministers, Rev. Charles C. Albertson ; The man of the medicine case, Annie May Cheney, M. D.; The patient, John B. Olmsted, Esq.; The conquest of medical superstition, Alice Ross Bennett, M. D.; Ninety-eight, John Livermore Hazen, M.D.

At a late hour the company separated, speaking words of praise for the delightful entertainment, all of which were well deserved by the committee of arrangements, headed by Dr. Albert T. Lytle as chairman.

